# Targeted treatment of metastatic castration-resistant prostate cancer with sipuleucel-T immunotherapy

**DOI:** 10.1007/s00262-015-1707-3

**Published:** 2015-05-30

**Authors:** Peter F. Mulders, Maria De Santis, Thomas Powles, Karim Fizazi

**Affiliations:** 1 Department of Urology, Radboud University Nijmegen Medical Centre, P.O. Box 9101, 6500HB Nijmegen, The Netherlands; 2 LBI-ACR VIEnna and 3rd Medical Department, Kaiser-Franz-Josef-Spital, Vienna, Austria; 3 Barts Cancer Institute, Queen Mary University of London, London, UK; 4 Department of Cancer Medicine, Institut Gustave Roussy, University of Paris-Sud, Villejuif, France

**Keywords:** Immunotherapy, Metastatic castration-resistant prostate cancer, Prostate cancer, Sipuleucel-T

## Abstract

**Context:**

Prostate cancer remains highly prevalent and has a poor clinical outcome once metastatic. Sipuleucel-T is an autologous cellular immunotherapy approved for the treatment of metastatic castration-resistant prostate cancer (mCRPC). Sipuleucel-T treatment extends survival but is independent of traditional short-term markers of treatment response observed with chemotherapy and contemporary hormonal treatments. Therefore, it is essential that clinicians understand the mechanism of action of sipuleucel-T and how this can translate in the clinic.

**Objective:**

This article aims to summarize the current knowledge of sipuleucel-T therapy and its effects in mCRPC.

**Evidence acquisition:**

Relevant publications describing sipuleucel-T clinical data and information relating to immunotherapies were identified.

**Evidence synthesis:**

Treatment with sipuleucel-T extends survival, with side effects being usually mild or moderate and manageable within the outpatient setting. The long-term immune responses generated by sipuleucel-T correlate with a survival benefit. Sipuleucel-T shows a greater magnitude of clinical benefit when used in patients earlier in the mCRPC setting.

**Conclusions:**

Sipuleucel-T stimulates long-lived immune responses that translate into long-term clinical benefit. The treatment course (three infusions at weeks 0, 2, and 4) is associated with manageable side effects. Short-term markers of future benefit would be clinically useful, and information on effective treatment combinations or sequences is awaited.

**Patient summary:**

Sipuleucel-T treatment directs the patient’s own immune system to target and remove prostate cancer cells and increases life expectancy. Patients whose cancer is less advanced generally have a more ‘active’ immune system and may benefit the most from this treatment.

## Introduction

Approximately 400,000 men are diagnosed with prostate cancer in Europe each year, equating to almost a quarter of all male cancers [1]. Around 90,000 prostate cancer-related deaths occur annually in Europe, giving it the third highest mortality rate after lung and colon cancers [1], and in the USA, it is surpassed only by lung cancer [2].

Men with localized prostate cancer are generally treated with active surveillance, brachytherapy, surgery, or radiotherapy with or without androgen deprivation therapy (ADT), depending on the individual prognosis [3]. Patients treated with ADT alone for metastatic disease usually respond well, but ultimately biochemical relapse and disease progression may occur. Castration-resistant prostate cancer (CRPC) may be suspected in patients with new symptoms, rising prostate-specific antigen (PSA) levels, or other new evidence of disease while being treated with ADT [4]. Treatment options for patients who progress to metastatic CRPC (mCRPC) have evolved dramatically over recent years and include the following: immunotherapy with sipuleucel-T [5, 6]; androgen biosynthesis inhibition with abiraterone acetate and prednisone [7]; androgen signaling inhibition with enzalutamide [8]; chemotherapy in combination with steroids, such as docetaxel or cabazitaxel and prednisone; and bone-targeted agents including the radioisotope radium-223 dichloride [9]. Additional immunotherapies are under investigation for mCRPC, such as the cytotoxic T-lymphocyte antigen 4 inhibitor ipilimumab, for which phase III trial results were recently published [10].

Sipuleucel-T is an autologous cellular immunotherapy that is indicated for the treatment of asymptomatic or minimally symptomatic mCRPC [11]. In April 2010, sipuleucel-T became the first US Food and Drug Administration (FDA)-approved immunotherapy following the demonstration of significant overall survival (OS) improvement in patients with primarily asymptomatic or minimally symptomatic mCRPC [5, 12, 13]. Sipuleucel-T was also recently approved by the European Medicines Agency (EMA) for the treatment of asymptomatic or minimally symptomatic metastatic (non-visceral) CRPC in male adults in whom chemotherapy is not yet clinically indicated [14]. A European study of sipuleucel-T is ongoing across centers in Vienna, Nijmegen, London, and Paris, with the product manufactured in Maastricht (The Netherlands) using the standard methodology (see section ‘Manufacture of sipuleucel-T’). In addition, sipuleucel-T is being explored in earlier and later settings, and the initial findings have been positive [15]. In clinical trials, sipuleucel-T was well tolerated and had manageable side effects [16]; the majority of adverse events were mild or moderate and could be managed in an outpatient setting. In order to appreciate the long-term clinical benefits of sipuleucel-T treatment, it is important to understand how these are derived from its mode of action.

## Evidence acquisition

Relevant publications presenting data on sipuleucel-T treatment, predominantly from key clinical trials, were identified. Additional articles commenting on these data, and on the results achieved with sipuleucel-T and other immunotherapies, were also included.

## Evidence synthesis

Data and other information from the identified publications were collated and are presented below.

### Manufacture of sipuleucel-T

Sipuleucel-T uses the patient’s own cells to stimulate an immune response against prostatic acid phosphatase (PAP), which is an appropriate target as it is expressed almost exclusively by prostate cells. The potential for inappropriate immune reactions is limited because sipuleucel-T is composed of the patient’s own cells.

**Fig. 1:**
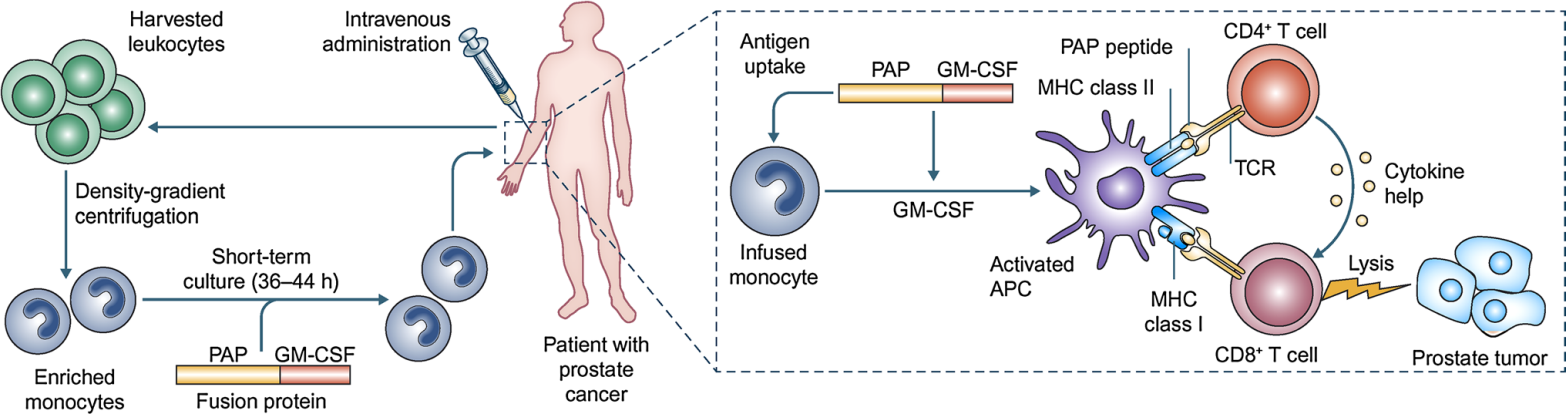
Sipuleucel-T immunotherapy and proposed mode of action. Leukocytes are harvested from the patient by leukapheresis and sent for processing, in which monocytes are enriched by density-gradient centrifugation. The monocytes are incubated with the PA2024 fusion protein of PAP and GM-CSF. PA2024 is taken up by immature APCs, such as DCs, and enhances their maturation. The resulting ‘product’ is returned to the clinic and administered intravenously to the patient. Theoretically, the transfused APCs will present PAP-derived peptides to the host immune system in vivo, activating CD4^+^ and CD8^+^ T cells and initiating adaptive immune responses. Adapted by permission from Macmillan Publishers Ltd: Nature Reviews Immunology 2010, Vol. 10, Drake CG, Prostate cancer as a model for tumor immunotherapy, pp. 580–93, copyright 2010 [17]. *APC* antigen-presenting cell. *CD* cluster of differentiation. *DC* dendritic cell. *GM*-*CSF* granulocyte–macrophage colony-stimulating factor. *MHC* major histocompatability complex. *PA2024* fusion protein of PAP and GM-CSF. *PAP* prostatic acid phosphatase*. TCR* T cell receptor

The first step in the manufacture of sipuleucel-T is to isolate the patient’s peripheral blood mononuclear cells (PBMCs), including antigen-presenting cells (APCs) such as dendritic cells, macrophages, and B cells, using leukapheresis (Fig. 1). These cells are then cultured in vitro for 36–44 h with a fusion protein, PA2024, composed of recombinant PAP fused to granulocyte–macrophage colony-stimulating factor (GM-CSF), a cytokine that stimulates APCs. Stimulating APCs ex vivo is thought to be an effective approach for initiating an immune response away from the potentially immunosuppressive effects of the prostate tumor. Ex vivo PA2024-activated APCs have been shown to process and display fragments of the PAP moiety on the cell surface [18]. The sipuleucel-T product is then reinfused back into the patient, where the activated APCs stimulate an immune response against PAP and consequently against the cancerous prostatic cells. The potency of sipuleucel-T may be assessed by the upregulation of cluster of differentiation 54 (CD54) on APCs (measured as an increase in the ratio of the average number of molecules of CD54 on APCs pre- vs post-culture with PA2024) [18]. Cumulative APC activation and APC number correlated with OS in an analysis of three randomized controlled phase III trials [19]. Thus, current sipuleucel-T manufacture requires that the product contains a minimum of 50 million autologous CD54^+^ cells [11]. The complete treatment involves three cycles 2 weeks apart (weeks 0, 2, and 4), with each cycle comprising fresh leukapheresis, cell isolation and culture with PA2024, and reinfusion. The sipuleucel-T product contains T cells, APCs, natural killer cells, and B cells, and the cellular composition does not change between treatment weeks nor between pre-culture compared with the final product [19].

### Engagement of the immune system during manufacture of sipuleucel-T

**Fig. 2:**
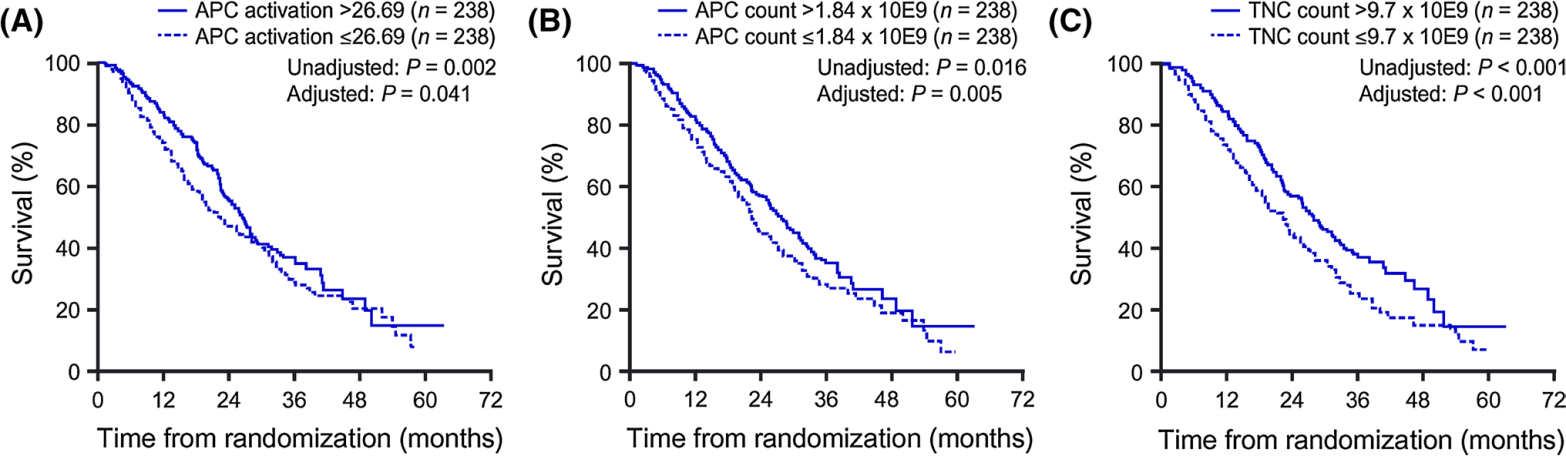
Significant correlation between OS and cumulative **a** APC activation, **b** APC (CD54^+^ cell) count, and **c** TNC count (product parameters) following co-culture of cells with PA2024. Kaplan–Meier survival plots are shown for each product parameter above versus below the median value; *p* values from analysis of each parameter as a continuous measure were calculated with and without adjustment for baseline PSA and LDH levels. Reproduced from Cancer Immunology Immunotherapy 2013, Vol. 62, Sheikh NA, Petrylak D, Kantoff PW, et al., Sipuleucel-T immune parameters correlate with survival: an analysis of the randomized phase 3 clinical trials in men with castration-resistant prostate cancer, pp. 137–47. Copyright 2012, the Author(s) [19]. *APC* antigen-presenting cell, *CD* cluster of differentiation, *GM-CSF* granulocyte-macrophage colony-stimulating factor, *LDH* lactate dehydrogenase, *OS* overall survival, *PA2024* fusion protein of PAP and GM-CSF, *PAP* prostatic acid phosphatase, *PSA* prostate-specific antigen, *TNC* total nucleated cell

Significant levels of in vitro APC activation, measured by CD54 upregulation, are induced during co-culture of the patient’s cells with PA2024 [18–20]. Greater increases in APC activation are seen during the second and third (weeks 2 and 4) in vitro co-cultures compared with the first (week 0). This response profile is akin to that generated by traditional vaccines—a prime-boost effect is observed after the first dose when administering sequential vaccinations. Significant positive correlations were observed between OS and cumulative (across the three infusions) post-culture in vitro APC activation, APC count, and total nucleated cell (TNC) count, which remained after adjusting for baseline PSA and lactate dehydrogenase (LDH) levels (Fig. 2) [19].

There is evidence to suggest that antigen-specific T and B cell responses developed following the first sipuleucel-T infusion, and that these were re-stimulated during the subsequent co-cultures. Cytokines that are associated with T cell activation were detected in culture medium from the second and third in vitro co-cultures, and included interleukin (IL)-2, IL-4, IL-5, IL-6, IL-10, IL-13, IL-17, interferon gamma (IFNγ), IFNγ-inducible protein 10, and tumor necrosis factor alpha [19, 20]. In addition, levels of the T cell activation markers CD134 and CD137 were increased on CD4^+^ and CD8^+^ T cells after culture with PA2024, and there was evidence of B cell maturation and activation [20]. Furthermore, PA2024-specific T cell ‘recall’ responses, measured by antigen-specific proliferation and IFNγ production, could be detected in pre-culture cells from the second and particularly the third leukapheresis [19, 20]. This suggests that functional T cell responses were generated in the body after the first infusion and were subsequently boosted after the second infusion. Each of these findings reinforces the hypothesis that sipuleucel-T treatment induces an immunologic ‘prime-boost’ response.

### Post-treatment immune responses with sipuleucel-T and correlation with OS

T cell responses, such as proliferation and IFNγ production in response to stimulation with either PA2024 or PAP, could be detected in PBMCs from the majority of patients treated with sipuleucel-T [19]. This indicates that the treatment generated prostate antigen-specific immune responses. Preliminary evidence also suggests that this peripheral blood immune response has a direct effect within the prostate, as demonstrated by an increased number of leukocytes around the tumor site [21]. In the neoadjuvant setting, patients with non-metastatic prostate cancer who were treated with sipuleucel-T displayed a ≥3-fold increase in mean cells/area at the tumor interface for total T cells, CD4^+^ helper T cells, and CD8^+^ cytotoxic T cells, compared with pre-treatment biopsy or with non-interface areas of benign or malignant tissue [21].

Robust post-treatment antibody responses specific to PA2024 and PAP have also been detected in the majority of patients treated with sipuleucel-T, but not in control patients [19]. In the majority of sipuleucel-T-treated patients, antigen-specific antibodies remained present at detectable levels in serum for at least 26 weeks. The majority of the antibody responses were initially immunoglobulin M, with immunoglobulin G antibodies emerging 2–4 weeks after the last infusion. This isotype switching is consistent with vaccine-induced immunologic memory. In addition, anti-tetanus antibody responses were unchanged after sipuleucel-T treatment, indicating that bystander immune responses were not generated.

**Fig. 3:**
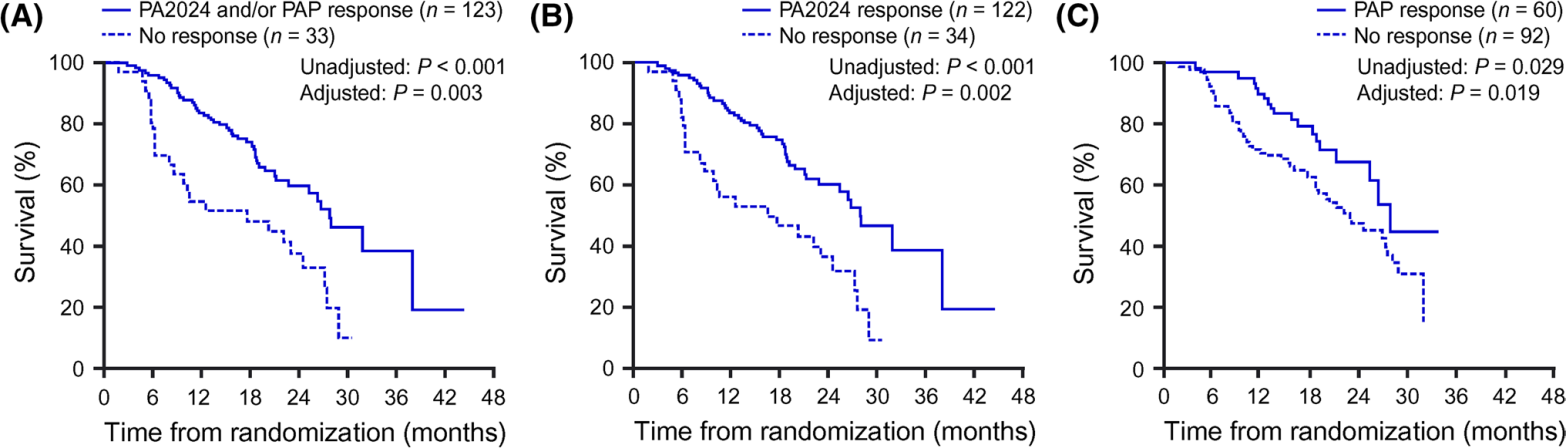
Significant correlation between OS and immune responders or non-responders to sipuleucel-T treatment as determined by **a** response to either PA2024 or PAP, **b** response to PA2024, and **c** response to PAP, in at least one of the three immune response assays (antibodies, IFNγ-production, and proliferation). Kaplan–Meier survival plots are shown; *p* values from analysis of each parameter were calculated with and without adjustment for baseline PSA and LDH levels. Reproduced from Cancer Immunology Immunotherapy 2013, Vol. 62, Sheikh NA, Petrylak D, Kantoff PW, et al., Sipuleucel-T immune parameters correlate with survival: an analysis of the randomized phase 3 clinical trials in men with castration-resistant prostate cancer, pp. 137–47. Copyright 2012, the Author(s) [19]. *GM*-*CSF* granulocyte–macrophage colony-stimulating factor*. IFN*γ interferon gamma. *LDH* lactate dehydrogenase. *OS* overall survival*. PA2024* fusion protein of PAP and GM-CSF. *PAP* prostatic acid phosphatase. *PSA* prostate-specific antigen

Antibodies in the systemic circulation may, theoretically, reach many tissues, potentially allowing the destruction of malignant cells throughout the body and resulting in long-term, widespread immunosurveillance. There is also potential for the immune system to recognize additional ‘nontarget’ cancer antigens through a process known as epitope spreading, to enable it to better identify and remove cancerous cells. Epitope spreading is particularly noted in the development of long-term antibody responses. This antigen cascade event may explain why OS significantly correlated with an antibody response against PA2024 [hazard ratio (HR) 0.42; *p* < 0.001] [19] and highlights the importance of humoral immunity in this context. OS also significantly correlated with the development of either a functional T cell response (above) or a humoral response (Fig. 3).

Overall, these data support the concept that engagement of key immune cell types (APCs, T, and B cells) during sipuleucel-T treatment contributes to the OS benefit. However, there are additional data to suggest that engagement of ‘less prominent’ immune cell types could also be important. Approximately 30 % of the patients in three phase III trials experienced a transient increase in peripheral blood eosinophil counts from week 6 to week 14, directly after completion of sipuleucel-T treatment [22]. Eosinophils are often considered to be rare, cytotoxic cells that are only relevant in parasitic infections and asthma [23]. However, there is increasing evidence that eosinophils may be involved in the generation of sipuleucel-T antitumor immune responses [23]. This may be through interactions with T cells (recruitment and polarization or direct antitumor activity), B cells (antibody responses), or dendritic cells (activation/recruitment). Indeed, the transient, elevated eosinophil counts in patients receiving sipuleucel-T correlated with OS, prostate cancer-specific survival, and antigen-specific humoral responses [22].

### Optimizing the use of sipuleucel-T

#### Surrogate markers for OS

Unlike traditional chemotherapy, sipuleucel-T treatment is associated with a long-term OS benefit but not a reduction in time to objective disease progression (defined by at least one of the following criteria: an increase in at least 50 % in the sum of the products of diameters for index lesions; the new appearance or unequivocal progression of non-index lesions; at least two new lesions on bone scanning; and/or a new pathologic fracture or spinal cord compression [5]). The same phenomenon has been noted with other immunotherapies. For example, treatment with PSA-TRICOM did not increase time to prostate cancer progression but achieved a significant improvement in 3-year OS {30 % vs. 17 %; HR 0.56 [95 % confidence interval (CI), 0.37–0.85]} and median survival (25.1 vs. 16.6 months; *p* = 0.0061) versus control [6]. Similarly, in melanoma, ipilimumab treatment did not result in an improvement in median progression-free survival (PFS), but a significant extension of median OS was achieved (10.1 months in ipilimumab arms) compared with active control (6.4 months; *p* < 0.001 and *p* = 0.003, respectively) [24]. Therefore, traditional short-term indicators of long-term survival benefits may not be appropriate with sipuleucel-T or other immunotherapies.

**Fig. 4:**
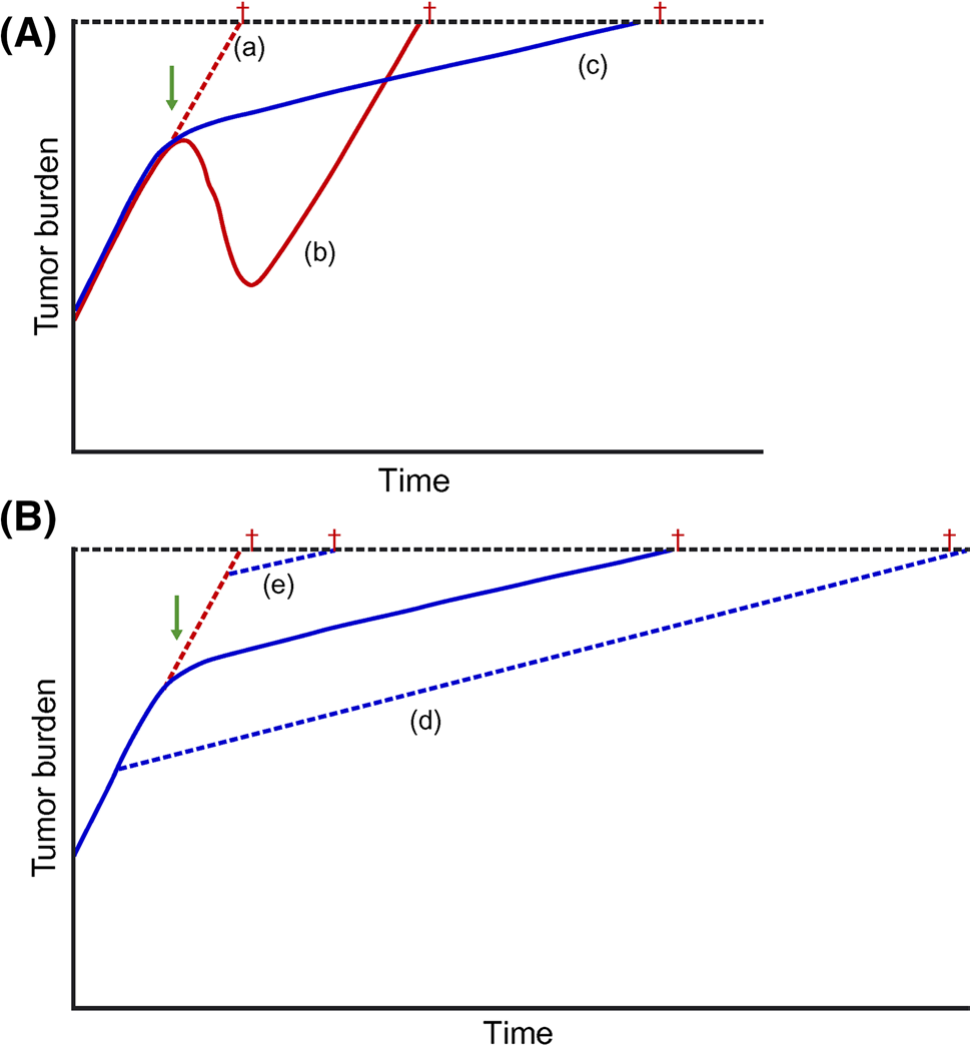
Proposed kinetics of immunomodulatory treatments. In **a,** immunotherapy is compared with cytotoxic chemotherapy. Tumor burden is shown if *a* no therapy is initiated, *b* chemotherapy is initiated, or *c* immunomodulatory therapy is initiated. For patients who received immunotherapy, there may be little if any reduction in tumor size, and therefore little or no increase in time to progression, but an increase in OS. *Dagger* denotes time of death. In **b,** early versus late initiation of immunomodulatory therapy is explored. The survival benefit may be increased if immunomodulatory treatment is initiated earlier in disease progression (*d*) but may be decreased in patients with a large tumor burden (*e*). Reproduced from Schlom J. Therapeutic cancer vaccines: current status and moving forward. J Natl Cancer Inst 2012;104(8):599–613, by permission of Oxford University Press [31]. *OS* overall survival

This seeming discrepancy may relate to the immunomodulatory mode of action. Antigen-specific immune responses take time to generate, and therefore tumor shrinkage may be delayed compared with traditional therapies (Fig. 4a) [25, 26]. It is also likely that an initial inflammatory influx of leukocytes into a tumor could lead to an initial, nonpathogenic increase in size, followed by a subsequent, long-term antitumor response [25]. Thus, immunologic memory could enable the body to continue to fight the tumor and slow its growth long after the immunotherapeutic treatment has been completed [25, 26]. Furthermore, theoretically, long-term immunosurveillance could act throughout the body as well as within the prostate, and could potentially target metastatic cells, limiting their spread.

Clinically, short-term markers of future benefit from immunotherapies would be very useful. In this regard, the transient increase in eosinophil counts in peripheral blood, noted from week 6 to week 14, may play a role given that this correlates with OS [22]. Eosinophil counts are routinely measured in this context; therefore, after further investigation and validation, elevated eosinophil counts could be a potentially useful surrogate marker.

#### Sipuleucel-T’s position within the treatment paradigm

Sipuleucel-T was evaluated in three randomized phase III studies [D9901: NCT00005947; D9902A: NCT01133704; and Immunotherapy for Prostate Adenocarcinoma Treatment (IMPACT; D9902B): NCT00065442] of patients with asymptomatic or minimally symptomatic mCRPC, and consistent improvements in OS, compared with placebo, were observed across the studies [5, 12, 13, 16]. FDA and EMA approval of sipuleucel-T was based primarily on the pivotal IMPACT study which demonstrated that sipuleucel-T was associated with improved median survival (4.1 months; 25.8 vs. 21.7 months in the sipuleucel-T and control arms, respectively), increased 3-year survival (31.7 vs. 23.0 % in the sipuleucel-T and control arms, respectively) and a 22.5 % reduction in risk of death (HR 0.78; 95 % CI 0.61–0.98; *p* = 0.03). Although there were more adverse events in the sipuleucel-T group, compared with the placebo group, these events were primarily related to leukapheresis and infusion. Most events were mild to moderate (typically chills, fever, fatigue, nausea, and/or headache), occurred within 1 day of infusion, and resolved within 1–2 days, with very few patients (0.9 %) prematurely discontinuing sipuleucel-T therapy [5].

A commentary by Huber et al. [27] questioned the trial design and the validity of the results from the IMPACT study. The following concerns were raised: (1) unexpected interactions between patient age and survival, (2) older patients in the placebo group appeared to have shorter OS than might be expected based on the results of other studies, and (3) the number of cells harvested and reinfused to the patients. Three subsequent publications rebutted these concerns [28–30] and presented clear explanations as to why, due to comparisons between asymmetric and small subgroups, and a lack of evaluable data, the article by Huber et al. was misleading. Moreover, independent bodies, such as the FDA and the Center for Medicare and Medicaid Services, also found the issues raised by Huber et al. not to be credible, supporting the published rebuttals. In summary, the claims made by Huber et al. were considered to be unfounded, a view supported overwhelmingly by the regulatory bodies and key thought leaders in the field.

Further to the above-summarized clinical trial data supporting improvements in OS of patients with asymptomatic or minimally symptomatic mCRPC treated with sipuleucel-T, several lines of evidence support sipuleucel-T use earlier within the treatment paradigm. Treating patients earlier in the disease course would allow more time for the immune response to develop and would theoretically allow patients to benefit most from the long-term OS advantage (Fig. 4b). In addition, several factors associated with improved survival following sipuleucel-T treatment, such as APC activation and transient elevations in eosinophil counts, are greater or more frequent in patients with characteristics of earlier-stage disease [15, 22, 27]. Indeed, the cumulative fold increase in APC activation, as measured by CD54 upregulation, was significantly greater in patients with earlier-stage disease; the fold increase was 35.5 in neoadjuvant patients, 28.7 in asymptomatic or minimally symptomatic mCRPC patients, and 21.8 in mCRPC patients (*p* < 0.0001; Joncheere-Terpstra test) [31]. In addition, patients with characteristics of earlier-stage disease may show greater OS [32], a greater time to first opioid analgesic use, and a trend toward a delay in time to disease-related pain after sipuleucel-T treatment than those with later-stage disease characteristics [33].

### Future directions with sipuleucel-T in the management of prostate cancer

Several ongoing and planned studies will investigate further the optimal role for sipuleucel-T in the management of prostate cancer (Table 1). These trials are addressing several aspects of sipuleucel-T therapy, such as use in earlier treatment settings (neoadjuvant, hormone sensitive, and non-metastatic) and the combination of sipuleucel-T with other agents (chemotherapy, radiotherapy, ADT, abiraterone acetate, and immunotherapeutics). Use of sipuleucel-T earlier in the prostate cancer paradigm may have considerable benefits to patients, since these patients are generally able to mount more robust immune responses [15, 22, 27], and there may be more time for the sustained, durable immune responses to exert their clinical benefit [32–34]. Preliminary data on the use of sipuleucel-T before, concurrently with, or after additional anticancer therapies are also encouraging. In the IMPACT trial, sipuleucel-T use was associated with increased survival versus placebo irrespective of previous chemotherapy or docetaxel use, radiotherapy, prostatectomy, orchiectomy, castration, or combined androgen blockade [5]. In addition, a sipuleucel-T treatment effect was observed in patients with and without subsequent docetaxel use [35]. Preliminary data from the P11-3 study suggest that treatment with abiraterone acetate and prednisone does not substantially affect the leukocyte yield for most patients, and that sipuleucel-T can, therefore, be successfully manufactured alongside the use of these agents [36]. The potential effects of treatment sequencing on clinical outcomes are also under investigation. Preliminary interim results from the phase II STAND trial (sequencing of sipuleucel-T and ADT in men with non-metastatic prostate cancer; NCT01431391) show that combination treatment with ADT followed by sipuleucel-T may augment immune responses, which could potentially increase efficacy compared with either treatment alone  [37].

**Table 1:** Planned and ongoing^a^ sipuleucel-T clinical trials in prostate cancer

Patients and indication	Intervention(s) and study design	Phase	Planned enrollment	Outcomes		Current status^a^	Clinical trials. gov identifier
				Primary	Secondary		
Localized, untreated prostate cancer	Sipuleucel-T as neoadjuvant treatment	2	40	Immune responses	Other immunologic tests	Ongoing	NCT00715104
Non-metastatic prostate cancer	Sipuleucel-T started before or after ADT	2	60	Immune responses	Safety, immune responses, sipuleucel-T product parameters, PSA changes	Ongoing	NCT01431391
CRPC	Sipuleucel-T with or without a following pTVG-HP DNA vaccination	2	30	Immune responses	PFS, time to radiographic disease progression, PSA doubling time	Recruiting	NCT01706458
According to sipuleucel-T label	Sipuleucel-T followed by ipilimumab	1	9	Immune responses	PSA doubling time and % decline, time to PSA progression or salvage therapy	Recruiting	NCT01832870
According to sipuleucel-T label	Observational registry of patients treated with sipuleucel-T in routine clinical practice	–	1500	Cerebrovascular events	Survival	Recruiting	NCT01306890
mCRPC	Sipuleucel-T with or without radiation therapy	2	50	Patients receiving all sipuleucel-T doses	Safety, immune responses, radiologic responses	Planned	NCT01807065
mCRPC	Sipuleucel-T and SABR	2	41	Time to progression	Immune responses	Planned	NCT01818986
mCRPC	Sipuleucel-T with radiation therapy	Pilot study	15	Immune responses	Safety, PSA decline, OS, cancer-specific survival	Planned	NCT01833208
mCRPC	Sipuleucel-T before or after lymph node biopsy	1	20	Immune responses in lymph nodes	Serum antibody levels	Recruiting	NCT02036918
mCRPC	Sipuleucel-T with concurrent or sequential enzalutamide	2	100	Immune responses	Time to PSA progression, OS, safety, magnitude of immune responses over time	Recruiting	NCT01981122
mCRPC	Sipuleucel-T, alone or with CT-011 (anti-PD1 antibody), cyclophosphamide, or both	2	57	Feasibility and immune responses	PFS and OS	Recruiting	NCT01420965
mCRPC	Sipuleucel-T manufactured at a European facility	2	45	Sipuleucel-T product parameters	Safety	Recruiting	NCT01477749
mCRPC	Sipuleucel-T followed by indoximod or placebo	2	50	Immune responses	PCWG2 response rate, PFS, OS, QoL	Recruiting	NCT01560923
mCRPC	Concurrent versus sequential treatment with sipuleucel-T and abiraterone acetate	2	60	Cumulative CD54 upregulation	Safety, immune responses, sipuleucel-T product parameters	Ongoing	NCT01487863
mCRPC	Sipuleucel-T	2	80	Immune responses	Safety	Ongoing	NCT00901342
mCRPC	Sipuleucel-T with different concentrations of PA2024	2	120	Cumulative CD54 upregulation ratio	Immune responses, OS	Ongoing	NCT00715078
mCRPC	Sipuleucel-T followed by immediate or delayed ipilimumab	2	66	Safety, impact of timing of ipilimumab	PSA decline, radiographic response, immune responses	Ongoing	NCT01804465
mCRPC previously treated with sipuleucel-T	Sipuleucel-T	2	90	Immune response	Safety, OS	Recruiting	NCT01338012

^a^According to a search of www.clinicaltrials.gov (search terms ‘sipuleucel-T OR Provenge’) on April 29, 2014. Only relevant trials were included, and completed studies were excluded

*ADT* androgen deprivation therapy; *CD* cluster of differentiation; *CRPC* castration-resistant prostate cancer; *DNA* deoxyribonucleic acid; *GM*-*CSF* granulocyte-macrophage colony-stimulating factor; *mCRPC* metastatic castration-resistant prostate cancer; *OS* overall survival; *PA2024* fusion protein of PAP and GM-CSF; *PAP* prostatic acid phosphatase; *PCWG2* Prostate Cancer Working Group 2; *PD* programmed cell death; *PFS* progression-free survival; *PSA* prostate-specific antigen; *QoL* quality of life; *SABR* stereotactic ablative body radiation

## Conclusions

New, effective drugs with different mechanisms of action are essential for the treatment of prostate cancer, which remains highly prevalent and has both a high mortality rate and poor clinical outcome after biochemical relapse. Sipuleucel-T is a significant advance in the treatment of mCRPC and provides clinicians with an additional effective therapy. Treatment with sipuleucel-T extends survival and is associated with easily manageable side effects, which are usually mild. Few patients prematurely discontinue sipuleucel-T therapy, and substantial long-term benefits can be achieved with just a 4-week treatment course. Research is ongoing to identify short-term markers of the long-term clinical benefits of sipuleucel-T treatment, to investigate its use in earlier disease settings, and in combination with other therapies.

## Abbreviations

ADT: Androgen deprivation therapy
APC: Antigen-presenting cell
CD: Cluster of differentiation
CI: Confidence interval
CRPC: Castration-resistant prostate cancer
DC: Dendritic cell
DNA: Deoxyribonucleic acid
EMA: European Medicines Agency
FDA: US Food and Drug Administration
GM-CSF: Granulocyte–macrophage colony-stimulating factor
HR: Hazard ratio
IFNγ: Interferon gamma
IL: Interleukin
LDH: Lactate dehydrogenase
mCRPC: Metastatic castration-resistant prostate cancer
MHC: Major histocompatability complex
OS: Overall survival
PAP: Prostatic acid phosphatase
PBMC: Peripheral blood mononuclear cell
PD: Programmed cell death
PGWG2: Prostate Cancer Working Group 2
PFS: Progression-free survival
PSA: Prostate-specific antigen
QoL: Quality of life
SABR: Stereotactic ablative body radiation
TCR: T cell receptor
TNC: Total nucleated cell
